# Influence of CYP2C19*2/*17 genotype on adverse drug reactions of voriconazole in patients after allo-HSCT: a four-case report

**DOI:** 10.1007/s00432-017-2357-y

**Published:** 2017-02-28

**Authors:** Sienkiewicz Beata, Urbaniak-Kujda Donata, Dybko Jarosław, Wróbel Tomasz, Wiela-Hojeńska Anna

**Affiliations:** 1 Department of Clinical Pharmacology, Faculty of Pharmacy, Wrocław Medical University, 211a Borowska St., 50-556 Wrocław, Poland; 2 Department and Clinic of Haematology, Blood Neoplasms, and Bone Marrow Transplantation, Wrocław Medical University, 4 Wybrzeże Pasteura St., 50-367 Wrocław, Poland

**Keywords:** CYP2C19*2/*17, Voriconazole, Adverse drug reactions, Genotype, Hematology

## Abstract

**Purpose:**

Voriconazole (VCZ) is a new-generation triazol antifungal agent. CYP2C19 mutations have been reported to cause variability in VCZ pharmacokinetics, and thus lead to undesirable effects of pharmacotherapy. We observed four Caucasian patients who underwent allogenic hematopoietic stem cell transplantation, treated with voriconazole for prevention of fungal infections, to establish the impact of CYP2C19*2/*17 genotype on side effect occurrence.

**Methods:**

Genetic testing for CYP2C19 allele*2 and*17 was performed using two PCR-RFLP methods established by Goldstein and Blaisdell, and Sim et al. All four patients presented CYP2C19*2/*17 genotype.

**Results:**

The patients suffered from gastrointestinal, dermatological, neurological, hepatobiliary and renal adverse drug reactions (ADR). ADR could be best described by the use of VCZ. Other drugs potentially causing side effects were also taken into consideration. The presented complications were temporary and did not force dosage regimen adjustments or discontinuation of pharmacotherapy. After 2 months, the patients were discharged from hospital.

**Conclusions:**

Drug–drug interactions and ADR may occur even if an agent is used for prophylaxis only. We, therefore, should use any available tools for pharmacotherapy optimization—also genetic testing.

## Introduction

Voriconazole (VCZ) is a new-generation triazol antifungal agent. The drug is approved mainly for treatment of invasive aspergillosis, candidiasis and infections caused by *Scedosporium spp*. and *Fusarium spp*. (SPC Vfend 2016). As it is more effective in the prevention of mold infections than fluconazole (FCZ) and itraconazole, VCZ is also used in prophylaxis of invasive fungal infections (IFI) among immunocompromised patients undergoing allogenic hematopoietic stem cell transplantation (allo-HSCT) (Xu et al. 2013).

The drug is metabolized in the liver by CYP2C19, CYP3A4 and CYP2C9. Only mutations of the first isoenzyme have been reported to cause variability in voriconazole pharmacokinetics (Moriyama et al. 2015). This is why many scientists postulate the need for CYP2C19 genotyping for therapeutic drug monitoring of VCZ (Trubiano et al. 2014).

The concomitant use of other medicines in the therapy of haematological malignancies leads to drug–drug interactions. Especially the co-administration of voriconazole with phenytoin, fluconazole or cyclosporine A is reported to cause adverse effects due to changes in pharmacokinetic parameters (Purkins et al. 2003; Damle et al. 2011, SPC Sandimmun 2013).

In this paper, we present the case of more severe adverse drug reactions of VCZ in four patients with CYP2C19*2/*17 genotype after allo-HSCT and a concomitant drug–drug interaction analysis.

## Case reports

### Anamnesis

Four Caucasian patients, two males (aged 62 and 50) and two females (aged 61 and 63), with normal BMI were admitted to the Department and Clinic of Haematology, Blood Neoplasms, and Bone Marrow Transplantation for allogenic hematopoietic stem cell transplantation. The primary diseases were acute myeloid leukemia (AML) in two cases, myelodysplastic syndrome (MDS) and acute lymphoblastic leukemia (ALL). Three patients were treated with reduced-intensity conditioning (RIC) and one, suffering from ALL, with myeloablative conditioning (MAC). The treatment protocols are described below.

During RIC, mega-dose chemotherapy was performed using fludarabine I.V. on days −6 to −2, busulfan I.V. on days −5 to −2 inclusive and thymoglobulin I.V. on days −3 to −1. For MAC, the regimen consisted of TBI (2 × 2.0 Gy/day) and cyclophosphamide I.V. The patients received graft versus host disease (GVHD) prophylaxis combining cyclosporine A (CyA) I.V. with methotrexate I.V. To avoid infections, a co-medication with trimethoprim (TMP) *p.o*., acyclovir (ACV) *p.o*., and fluconazole (FCZ) *p.o*. was started. The decontamination was performed using ciprofloxacin *p.o*. Three days before transplantation, ACV and FCZ administrations have been suspended and voriconazole I.V. with ganciclovir I.V. was introduced into therapy as IFI and viral infection prophylaxis. After allo-HSCT, omeprazole I.V. was included as ulcer prevention. The dose regiments were established according to the particular summaries of product characteristics (SPC).

### Results

During VCZ pharmacotherapy, we observed adverse drug reactions (ADR) in frequency that was not noticed in other patients treated with the same protocols. One female demonstrated nausea and dizziness. The second one suffered from vomiting, constipation, rush, exfoliative dermatitis, vertigo, ataxia and aphasia. Among male patients, elevation of GGT (gamma-glutamyl transpeptidase) and in one male nausea, vomiting, rush, erythema, pneumonia, nephritis as well as hematuria were observed.

We performed genetic testing for CYP2C19 allele *2 and *17 determination, using two PCR-RFLP methods established by Goldstein and Blaisdell (1996) and Sim et al. (2006). All of the patients presented CYP2C19*2/*17 genotype. To our knowledge there is no definite information in the literature on the impact of this diplotype on voriconazole pharmacokinetic properties, but there is evidence suggesting a poor metabolizer (PM) phenotype, causing adverse drug reactions due to higher VCZ serum levels (Cervinski et al. 2013).

On the other hand, there are drug–drug interactions between voriconazole, cyclosporine A and omeprazole. This type of co-medication also leads to increased VCZ concentrations and is, therefore, a factor influencing ADR occurrence (SPC Sandimmun 2013; Wood et al. 2003).

### Treatment and progression

These presented complications were temporary and did not force dosage regimen adjustments or discontinuation of pharmacotherapy. After 2 months, the patients were discharged from hospital.

## Discussion

In the presented case report, adverse drug reactions of VCZ treatment occurred in patients with the CYP2C19*2/*17 diplotype, suggesting rather a poor metabolizer phenotype. The frequency of this genotype in the Caucasian population and the influence on voriconazole pharmacokinetic properties are unknown yet. On one hand, Weiss et al. (2009) reported that patients carrying this genetic variant demonstrated the same pharmacokinetic properties of VCZ as the ones with CYP2C19*1/*17 genotype. On the other hand, Chung et al. (2015) stated in their article that patients with CYP2C19*2/*17 variant were rather IM (intermediate metabolizers), but they also underlined the need for further research on this topic.

In another study conducted by Harmsze et al. (2012), the influence of CYP2C19 mutations on clopidogrel on-treatment platelet reactivity was established. Clopidogrel is a prodrug that needs to be metabolized to its active form. In patients demonstrating the CYP2C19*2/*17 genotype, the on-treatment platelet reactivity was increased compared with wild type, *1/*17 and *17/*17 diplotypes suggesting that CYP2C19*2/*17 genetic variant is associated with lower enzymatic activity. This may lead to the hypothesis that in the presented cases, the genotype led to decreased enzymatic activity, higher VCZ serum levels and as a result to its adverse reactions.

Another factor that has to be taken into account is the concomitant use of omeprazole. The drug is a competitive inhibitor of CYP2C19 isoenzyme. Its influence on the pharmacokinetic properties of voriconazole was established. Wood et al. (2003) showed that the co-administration of omeprazole leads to increased VCZ blood concentrations, what in case of our patients, considered to be PM, could have led to a greater elevation of voriconazole serum levels, and as a consequence to noticeable side effects (Boyd et al. 2012).

Certainly, the presented adverse effects may occur due to other drugs used in the patients’ treatment. Apart from gastrointestinal disturbances, such as nausea, vomiting and constipation, that are very frequent ADR during pharmacotherapy at all, hepatobiliary (GGT elevation), skin (exfoliative dermatitis, erythema), nervous system (ataxia) and renal disorders (nephritis) are undesirable effects common during VCZ treatment (SPC Vfend 2016).

Other observed ADR such as dizziness, vertigo, aphasia and pneumonia are more likely connected with methotrexate and cyclosporine A administration (SPC Sandimmun 2013, SPC Methotrexat-Ebewe 2016). This conclusion is based on the investigation of Mori et al. (2009) who demonstrated that voriconazole inhibits P-gp (P-glycoprotein) and increases CyA blood concentrations leading to adverse drug reactions, as cyclosporine A has a narrow therapeutic window, and the frequently noticed ADR during MTX treatment (SPC Methotrexat-Ebewe 2016).

## Conclusion

By reporting these cases, we would like to emphasize that drug–drug interactions and side effects may occur even if an agent is used for prophylaxis only. The definite identification of the medication causing ADR during polypharmacotherapy, e.g., accompanying allogenic hematopoietic stem cell transplantation, is challenging. Therefore, any available tools for pharmacotherapy optimization should be used including genetic testing.

In the presented case, previous determination of CYP2C19 mutation may have helped to avoid, at least partially, undesirable effects connected with voriconazole administration.
